# Trends, Variation, and Factors Influencing Antibiotic Prescribing: A Longitudinal Study in Primary Care Using a Multilevel Modelling Approach

**DOI:** 10.3390/antibiotics11010017

**Published:** 2021-12-24

**Authors:** Peter Devine, Maurice O’Kane, Magda Bucholc

**Affiliations:** 1 School of Computing, Engineering & Intelligent Systems, Ulster University, Derry BT48 7JL, UK; p.devine@ulster.ac.uk; 2 Altnagelvin Area Hospital, Western Health and Social Care Trust, Derry BT47 6SB, UK; maurice.okane@westerntrust.hscni.net

**Keywords:** antibiotics prescribing, antibacterial drugs, antimicrobial resistance, deprivation, primary care, general practice, multilevel modelling, mixed-effects model

## Abstract

Antimicrobial resistance has become one of the greatest threats to global health. Over 80% of antibiotics are prescribed in primary care, with many prescriptions considered to be issued inappropriately. The aim of this study was to examine the association between prescribing rates and demographic, practice, geographic, and socioeconomic characteristics using a multilevel modelling approach. Antibiotic prescribing data by 320 GP surgeries in Northern Ireland were obtained from Business Services Organisation for the years 2014–2020. A linear mixed-effects model was used to identify factors influencing antibiotic prescribing rates. Overall, the number of antibacterial prescriptions decreased by 26.2%, from 1,564,707 items in 2014 to 1,155,323 items in 2020. Lower levels of antibiotic prescribing were associated with urban practices (*p* < 0.001) and practices in less deprived areas (*p* = 0.005). The overall decrease in antibacterial drug prescriptions over time was larger in less deprived areas (*p* = 0.03). Higher prescribing rates were linked to GP practices located in areas with a higher percentage of the population aged ≥65 (*p* < 0.001) and <15 years (*p* < 0.001). There were also significant regional differences in antibiotic prescribing. We advocate that any future antibiotic prescribing targets should account for local factors.

## 1. Introduction

Antimicrobial resistance (AMR), identified by the World Health Organisation as one of the top global public health threats, is largely driven by excessive and inappropriate prescribing [1]. Increased AMR not only endangers the efficacy of antibiotics but also leads to infection-related complications, prolonged illness, higher hospitalisation rates, and increased mortality [2]. By 2050, the global cost associated with AMR is predicted to amount to $100 trillion in lost GDP and account for up to 10 million extra deaths a year [3]. The European Centre for Disease Prevention and Control estimated that approximately 33,000 patients in Europe die each year due to antibiotic-resistant infections [4]. In the UK, antibiotic resistance can cause up to 12,000 deaths per year [3].

To address the threat posed by AMR in the UK, the first fully integrated five-year strategy for tackling AMR was published in 2013 to address the actions needed in key AMR areas including infection prevention and control, prescribing practice, education and public engagement, development of treatment and technologies, surveillance, and research [4]. One of its main objectives was to reduce the levels of inappropriate antimicrobial prescribing by 50% by 2020 [5]. The delivery of key components of this five-year national action plan was supported by the establishment of the English Surveillance Programme for Antimicrobial Utilisation and Resistance (ESPAUR) focusing on enhancing the surveillance of antimicrobial resistance, monitoring antibiotic prescribing, and supporting interventions aimed at improving prescribing process [6]. These actions resulted in an overall 9% reduction in antibiotic consumption in primary and secondary care over the period 2014–2018 [7]. Building on the achievements of the 2013–2018 AMR strategy, the UK’s 2019–2024 AMR National Action plan set the new, ambitious targets to further reduce antibiotic use and improve diagnostic support for appropriate prescription [8]. In Northern Ireland (NI), the Department of Health (previously the Department of Health, Social Services and Public Safety) published in 2012 a five-year Strategy for Tackling Antimicrobial Resistance (STAR) [9]. One of the outputs of this initiative was the publication of annual reports on surveillance of antimicrobial use and resistance. Furthermore, in 2019, the NI Department of Health announced the five-year action plan, complimenting the UK Action Plan, and outlining the NI’s specific contribution to containing and controlling AMR [10].

Concerns about AMR resulted in increased monitoring and evaluation of antibiotic prescribing, mainly in primary care. It is estimated that over 80% of all antibiotics are prescribed by general practitioners (GPs) [11] and inappropriate prescribing in primary care may account for 8.8–23% of all prescriptions [12]. Several studies investigated the impact of different demographic, geographic, socioeconomic, and psychological factors on prescribing rates [13,14,15,16,17,18]. It has been demonstrated that antibiotic prescribing was associated with GP practice setting and a higher proportion of patients with long-term conditions [17]. In addition, antibiotic prescribing was found to be higher in more deprived areas in England [13], Scotland [14], and Wales [15]. For instance, Covvey at al. [14] showed that the overall antibiotic prescription rates were 36.5% higher in the most deprived (Scottish Index of Multiple Deprivation) SIMD quintile, compared to the least deprived quintile. In fact, antibiotic prescribing rates were shown to be rising in the most deprived areas [13]. Other factors related to higher prescribing rates included greater GP practice size and households with lower income [17,18]. Furthermore, significant between-GP practice variation in prescribing was attributed to GPs characteristics and other factors related to the professional care-delivery system of antibiotics [19]. Higher antibiotic prescribing rates were reported among older clinicians [18], male [20], non-UK qualified [20], and those professionally involved with drug company representatives [21].

This is the first study to provide a comprehensive longitudinal analysis of antibiotics prescribing in Northern Ireland primary care using a multilevel modelling approach. Specifically, we aim to (1) evaluate temporal changes in antibiotic prescription; (2) identify the extent of between-GP variations in antibiotic prescribing; and (3) examine the impact of demographic, geographic, socioeconomic, and practice characteristics on antibiotic prescribing.

## 2. Materials and Methods

### 2.1. Study Design

This was a retrospective cohort study of antibiotic prescribing using routinely collected primary care prescribing data.

### 2.2. Setting

NHS primary care in Northern Ireland was the setting, including general practices (*n* = 320) that were operational throughout the study period, with antibiotic prescriptions dispensed from January 2014 to December 2020. Accordingly, 31 practices that closed and 1 practice that opened during the period under review were excluded from the analysis.

### 2.3. Antibiotic Prescribing Data

Individual-level primary care prescribing data were obtained from the Business Services Organisation (BSO) prescribing and dispensing information systems [22]. The dataset includes openly accessible prescribing data from the monthly files published by BSO, which contain information on total number of items prescribed by GPs or other non-medical prescribers within GP practices (e.g., nurses) in NI. Antibacterial drugs were identified using British National Formulary (BNF) codes, namely, chapter 5.1.

### 2.4. Factors Associated with Antibiotic Prescribing

A number of factors, previously shown to be associated with variation in prescribing, were selected from publicly available data. These variables were: deprivation, GP practice size (the number of registered patients), Local Commissioning Group (LCG), setting (urban/rural), patient demographics, and household income. Given that deprivation and patient demographics were not available at the GP practice level, we used the aggregate data collated at the super output area (SOA) level for the practice postcode. SOAs are geographical areas designed for the publication of the Census outputs. As such, area-level deprivation was assigned to each GP practice by linking the SOA code of a GP practice’s postal address and the NI Multiple Deprivation Measure (MDM) rank [23]. The MDM rank consists of seven distinct component domains, namely, (i) income deprivation, (ii) employment deprivation, (iii) health deprivation and disability, (iv) education, skills and training deprivation, (v) proximity to services, (vi) living environment and (vii) crime and disorder [23]. The MDM provides a mechanism for ranking the 890 SOAs in NI from the most deprived (rank 1) to the least deprived (rank 890). Demographic data at the SOA level were obtained from the Mid-Year NI-Level Population Estimates [24]. We specifically extracted the proportion of SOA population aged >65 years and <15 years given that previous studies reported the association between level of antibiotic prescribing and percentage of older and younger patients registered with a GP [17]. We also included in the analysis the proportion of the population aged ≥65 and <15 living in households whose equivalised income was <60% of the NI median. The urban/rural nature of the GP practice was determined using the Northern Ireland Neighbourhood Information Service (NINIS) [25]. Accordingly, 85 GP practices were categorised as rural and 235 as urban. Furthermore, each practice was located within LCGs, which, in Northern Ireland, covers the same geographical area as Health and Social Care (HSC) Trusts. Figures for the GP practice size were obtained from the BSO Family Practitioner Services (2018–2020) [26] and the NI Council for Voluntary Action (2014–2017) [27].

### 2.5. Statistical Analysis

To allow temporal and between-GP practice analysis of changes in antibiotic prescribing, the standardised antibiotic prescribing rate (per 1000 patients) was calculated by dividing the total number of antibacterial drugs (‘items’) prescribed in each year by the total number of registered patients. The descriptive statistics, including median (interquartile range [IQR]) were provided for each time point. The Shapiro–Wilk test was used to determine if the data deviate from a normal distribution.

Given that our research design was based on the repeated-measures data for individual GP practices that were organised in nested levels (clusters), the descriptive analyses were complemented by performing the multilevel modelling analysis. The implementation of a multilevel modelling approach is a suitable choice for studies with the same outcome variable, which is repeatedly measured over time in the same study subjects (here, GP practices). Note that such repeatedly measured data are not independent. Many statistical techniques for analysing longitudinal data assume independence of the measurements. However, repeated measurements taken from the same individual (such as, GP practice) are often correlated, and hence, not independent. By not taking into account this correlation, we can obtain biased estimates and invalid *p*-values. The use of multilevel modelling, specifically a linear mixed-effects model, in our study allowed us not only to accommodate nonindependence of repeated measurements but also draw subject-specific conclusions.

We first evaluated the appropriateness of using our multilevel modelling approach by testing an “unconditional model” including only the dependent variable (i.e., standardised antibiotic prescription rates) and the grouping variable (i.e., GP practice). Note that no predictor variables were entered at this stage. The unconditional model revealed significant between-GP practice variation (*p* < 0.05), hence supporting the case for multilevel modelling [28]. Since the use of multilevel model was appropriate, the following predictor variables were added to the model: time, MDM rank, LCG, the urban/rural nature of the GP practice, GP practice size, the percentage of the population aged ≥65 and <15 years, and the proportion of the population aged ≥65 and <15 living in households whose equivalised income was <60% of the NI median. Next, we performed the variable selection based on *p*-value-based stepwise deletion [28], with Akaike information criterion (AIC) chosen as the optimal model selection approach [29]. The final model included the effect of time, MDM rank, LCG, rurality, GP practice size, the percentage of the population aged ≥65 and <15 years, as well as the interaction term between time and: (i) LCG; (ii) MDM rank; (iii) the percentage of the population aged ≥65; and (iv) the urban/rural nature of the GP practice as fixed effects. Features that did not significantly improve model fit, including the proportion of the population aged ≥65 and <15 living in households whose equivalised income was <60% of the NI median as well as the interaction terms between other covariates and time were excluded from the analysis. Random intercepts were modelled for each GP practice. We confirmed that the addition of a random intercept significantly improved model fit (*p* < 0.001). Maximum likelihood estimates were obtained using the Nelder–Mead method. Data management was performed using Python, version 3.6.9, with analysis carried out using R, version 3.6.3.

## 3. Results

### 3.1. Temporal Changes and Between-Practice Variation in Antibiotic Prescribing

A total of 10,165,296 antibiotic prescription items were dispensed in primary care over the 7 year study period. The total number of antibiotic prescriptions and the number of registered patients together with the year-on-year percentage change are shown in Table 1. The number of antibacterial prescriptions (‘items’) dropped by 26.2%, from 1,564,707 items in 2014 to 1,155,323 items in 2020. The largest decrease of 18.7% in the number of prescribed items was observed between 2019 and 2020. This substantial decrease may in part be explained by the COVID-19 pandemic, which resulted in citizens staying at home more (and therefore being less exposed to infectious diseases other than COVID-19) and the decrease in GP appointments. From March 2020, the majority of GP surgeries in Northern Ireland ceased face to face appointments and moved to video or telephone consultations in an effort to minimise the risk of spread of infection to both patients and staff. It has been shown that the average number of GP appointments per week in Northern Ireland decreased by 19%, from 432 appointments per week in 2019 to 348 appointments per week in 2020 [30]. Girvan et al. [31] concluded that fewer GP appointments during the first wave of COVID-19 resulted in an overall reduction in antibacterial prescribing of 5.49% compared with the same period the previous year. The number of patients registered in the 320 GP practices grew over the period under observation, from 1,855,949 in 2014 to 1,999,095 in 2020, an increase of 9.3%.

**Figure 1:**
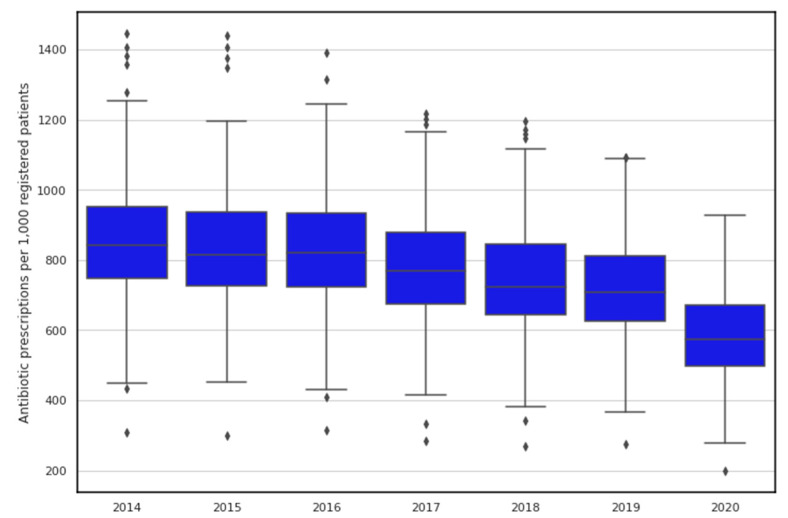
Temporal changes and variation in antibiotic prescribing rates in Northern Ireland. Horizontal line inside the box represents the median prescribing rate for each considered year while lower and upper extremes of whiskers act as interval boundaries of non-outliers. Each dot represents an outlier.

Figure 1 shows the trends and between-GP practice variation in the standardised number of antibiotic drugs (per 1000 patients). Although the general trend is downward, we can still observe extensive between-practice variation. Overall, the average standardised prescribing rate decreased from 843 items/1000 patients in 2014 to 578 items/1000 patients in 2020.

**Figure 2:**
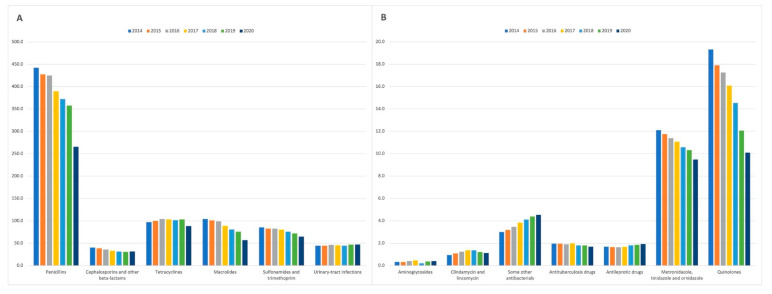
The standardised number of prescriptions for antibiotic classes (**A**) with the average annual prescribing rate >20 prescriptions per 1000 patients; (**B**) with the average annual prescribing rate <20 prescriptions per 1000 patients. Some other antibacterials include glycopeptide antibacterials, fidaxomicin, linezolid, polymixins, and rifaximin.

Figure 2A shows the changes in the standardised number of prescriptions for antibiotic classes with the average annual prescribing rate >20 prescriptions per 1000 patients. The three most common antibiotic classes, namely penicillins, macrolides, tetracyclines, reported a decrease in the standardised number of prescriptions over the study period, 40.0% (from 442.4 items/1000 patients in 2014 to 265.4 items/1000 patients in 2020), 45.4% (104.2 items/1000 patients in 2014 to 56.9 items/1000 patients in 2020), and 8.9% (97.0 items/1000 patients in 2014 to 88.4 items/1000 patients in 2020), respectively. Furthermore, there was a 22.2% decrease in prescribing rates for cephalosporins and other beta-lactams (from 40.1 items/1000 patients in 2014 to 31.2 items/1000 patients in 2020), and a 24.2% decrease in prescribing rates for sulphonamides and trimethoprim (from 85.3 items/1000 patients in 2014 to 64.6 items/1000 patients in 2020). The standardised number of prescriptions for urinary-tract infection drugs increased from 44.2 items/1000 patients in 2014 to 47.1 items/1000 patients in 2020 (an increase of 6.4%). Figure 2B presents the changes in the standardised number of prescriptions for seven antibiotic classes with the average annual prescribing rate <20 prescriptions per 1000 patients. Three of these classes reported a decrease in the standardised number of prescriptions over the study period, namely, antituberculosis drugs (a reduction of 13.78%); metronidazole, tinidazole and ornidazole (a reduction of 21.72%); and quinolones (a reduction of 47.75%). The increase in prescription rates was observed for aminoglycosides (24.24%), clindamycin and lincomycin (20.21%), antileprotic drugs (14.88%), and some other antibacterials including glycopeptide antibacterials, fidaxomicin, linezolid, polymixins, and rifaximin (51.33%). Supplementary materials: Table S1 provides the standardised number of prescriptions by antibiotic class in the period 2014–2020

Summary statistics of prescription data for the period 2014–2020 are presented in Table 2. The largest between-GP practice variation in antibiotic prescribing was observed in 2015 (range 297.9–1439.6 items per 1000 patients) and 2014 (range 308.6–1444.8 items per 1000 patients). The lowest between-practice variation in antibiotic prescribing was observed in 2020 (range 199.2–926.5 items per 1000 patients).

### 3.2. Factors Influencing Antibiotic Prescribing

Changes in standardised antibiotic prescribing rates were further evaluated using a linear mixed-effects model with a random intercept for each GP practice. We observed a substantial decrease in prescribing rates over time (β = −40.3, *p* < 0.001), i.e., for every one-year increase, the standardised number of prescribed drugs decreased by 40.3 items/1000 patients. On average, urban practices prescribed antibiotics less than rural practices (β = −65.4, *p* < 0.001). Practices located in areas with a lower level of deprivation (indicated by a higher score on the MDM Rank) were associated with a lower level of prescribing (β = −0.1, *p* = 0.005). A higher percentage of the population aged ≥65 years was associated with higher rates of prescribing (β = 9.9, *p* < 0.001), i.e., for one percentage increase in the proportion of the population aged ≥65, we observed the 9.9 items/1000 patients increase in the standardised number of antibacterial drugs. A higher percentage of the population aged <15 years was also associated with higher rates of prescribing (β = 11.0, *p* < 0.001). The GP practices located in Northern LCG (β = 103.8, *p* < 0.001), Western LCG (β = 125.3, *p* < 0.001), and Belfast LCG (β = 72.9, *p* = 0.001) prescribed, on average, more antibacterial drugs than GP practices in Southern LCG. The practice size (β = 0.64, *p* = 0.91) had no effect on prescribing rates.

Given the interaction terms in our model, we observed that the effect of time on antibiotic prescribing rates decreased by 0.007 items/1000 patients for every one-unit increase in MDM rank (β = −0.007, *p* = 0.03), meaning that the overall decrease in antibacterial drug prescriptions over time was larger in less deprived areas. Although higher percentage of the population aged ≥65 years was, on average, associated with higher rates of prescribing, this trend was becoming less significant over time (β = −0.38, *p* = 0.008). The effect of time on prescribing was also higher by 5.3 items/1000 patients (*p* = 0.003) for urban practices compared to rural practices. Finally, the change in the level of prescribing in GP practices located in Northern LCG, Western LCG, and South Eastern LCG was higher every year by 6.7 items/1000 patients (*p* = 0.003), 8.9 items/1000 patients (*p* < 0.001), and 9.7 items/1000 patients (*p* < 0.001), respectively, than prescribing in Southern LCG.

To further validate the robustness of the main findings, we implemented a sensitivity analysis to measure the potential impact of the COVID-19 pandemic on the association between different demographic, geographic, socioeconomic factors and antibiotic prescribing rates. Accordingly, a multilevel modelling analysis was conducted using only antibiotic prescription data from the period 2014–2019 (and thus, excluding the data collected during the COVID-19 pandemic), and the results compared with the findings of the main analysis based on the antibiotic prescription data from the period 2014–2020. The results of sensitivity analyses were consistent with those from the primary analysis. On average, urban practices prescribed antibiotics less than rural practices (β = −66.4, *p* < 0.001), and this difference was becoming more significant over time (β = 6.3, *p* = 0.001). Furthermore, lower levels of antibiotic prescribing were associated with GP practices in less deprived areas (β = −0.1, *p* = 0.007) as well as those in areas with higher percentages of the population aged ≥65 (β = 10.1, *p* < 0.001) and <15 years (β = 11.3, *p* < 0.001). Although higher percentage of the population aged ≥65 years was, on average, associated with higher antibiotic prescribing, this trend was becoming less significant over time (β = −0.42, *p* = 0.006). The GP practices located in Northern LCG (β = 104.7, *p* < 0.001), Western LCG (β = 128.9, *p* < 0.001), and Belfast LCG (β = 73.6, *p* = 0.002) prescribed, on average, more antibacterial drugs than GP practices in Southern LCG. Moreover, the change in the level of prescribing in GP practices located in Northern LCG, Western LCG, and South Eastern LCG was higher every year by 6.3 items/1000 patients (*p* = 0.009), 7.2 items/1000 patients (*p* = 0.007), and 7.5 items/1000 patients (*p* = 0.006), respectively, than prescribing in Southern LCG. The practice size (β = 0.36, *p* = 0.95) had no effect on antibiotic prescribing. Finally, for every one-year increase, the standardised number of prescribed antibiotics decreased by 28.4 items/1000 patients (*p* < 0.001). The annual rate of change in prescribing over the period 2014–2019 was therefore substantially lower than the 40.3 items/1000 patients decrease for every one-year increase in the period 2014–2020, highlighting the potential impact of the COVID-19 pandemic on the overall reduction in antibacterial prescribing.

## 4. Discussion

This is the first study to investigate determinants of antibiotic prescribing in Northern Ireland. We implemented a well-validated multilevel modelling approach allowing us to examine antibiotic prescribing practices in a systematic way and control for the potential confounding effects of practice, demographic, geographical, and socioeconomic factors. We showed a significant impact of several characteristics including rurality, deprivation, LCG, and percentage of the population aged ≥65 and <15 years on the standardised number of prescribed antibacterial drugs. We found that rural practices, practices in more deprived areas and those located in SOAs with high percentages of the population aged ≥65 and <15 years were associated with higher levels of antibiotic prescribing. In addition, we observed a large variation in antibiotic prescribing between LCGs and a significant effect of time on prescribing, given changes in MDM rank, rurality, and LCGs categories.

Our results showing that GP practices located in areas with a higher level of deprivation were associated with higher levels of antibiotic prescribing are in line with other studies [13,14,15]. Wise [13] reported a 20% difference in prescribing levels between the least and most deprived areas in England. More recently, Thomson et al. [32] showed that the most deprived areas of England had the highest levels of antibiotic prescribing. Covvey et al. [14]. demonstrated that the overall antibiotic prescription rates in Scotland were 36.5% higher in the most deprived quintile compared to the least deprived quintile, with the prescribing rates of 2.49 items and 1.58 items per 1000 patients per day, respectively. The analysis by Butler et al. [15] based on the data from 240 GP practices in Wales, showed an increase in the rate of antibiotic resistance, with levels 6% higher in the most deprived quartile. In Northern Ireland, higher rates of antibacterial prescribing were previously shown to be linked to increased deprivation, but this relationship was not adjusted for other covariates [33]. Given the practice size, previous studies reported no associations between single-handed practices and antibiotic prescribing rates [17,34,35].

Interestingly, our results also indicated that prescribing of antibacterial drugs decreased faster in areas with a lower level of deprivation. Furthermore, the observed patterns of antibiotic use by rurality and age were consistent with other studies [17,36,37,38]. For example, Curtis et al. [17] reported that GP practices with a higher proportion of patients >65 and <18 years old were more likely to prescribe more antibiotics. A study by Bou-Antoun et al. [39] found that antibiotic prescription rate per 1000 consultations was highest in adults aged 65 and older. We also observed a large between-practice variation in prescribing that was noted previously [16].

Our study has several limitations. First, we only assessed the changes and variation of antibiotic prescribing, but we did not evaluate the appropriateness of prescribing. Second, we acknowledge that characteristics, i.e., deprivation and demographics, that apply at an area level, do not necessarily apply at an individual practice level. Furthermore, our investigation was limited by the unavailability of GP practice-level datasets on patient (e.g., comorbidities) and GP characteristics (e.g., age, gender, training, workload, and attitude). The inclusion of GP characteristics could potentially further improve the fit of our model given the fact that significant between-GP practice variation in prescribing was previously related to intra-physician variability [16]. It is also possible that higher antibiotic prescribing observed in some GP practices was associated with higher proportions of registered patients being more susceptible to bacterial infections. Previous work showed that increased susceptibility for developing a bacterial infection, including measles [40], and tuberculosis [40], can be linked to nutritional deficiency [41], smoking [42], and reduced vaccination uptake [43].

The observed decrease in antibiotic prescribing in Northern Ireland has likely been influenced by the implementation of antimicrobial guidance for primary care that complements the work of HSC Trusts in reducing antimicrobial resistance [44]. Furthermore, the reduction in antibiotic prescribing might have been related to the employment of a large number of practice-based pharmacists in recent years who advise on antimicrobial prescribing, in particular, on the dose and duration of long-term and repeat prescriptions [10]. As a part of the Pharmaceutical Clinical Effectiveness (PCE) programme, GP surgeries are also supported by HSCB pharmacy advisors who provide GPs with practice-specific feedback (COMPASS reports) indicating not only how their prescribing rates differ from other practices but also identifying potential efficiencies [45]. Despite the reduction in antibiotic consumption over time, there is still large variability in prescribing among GP practices in Northern Ireland. This variation can be partially explained by demographic, geographical, and socioeconomic characteristics. Any antibiotic prescribing targets should therefore account for local factors so that GP practices are not inappropriately penalised.

## 5. Conclusions

Our findings contribute to the debate on determinants of antibiotic prescribing in primary care and provide actionable insights to policy makers responsible for antimicrobial stewardship and public health campaigns. Indicators of antibiotic prescribing used in NI primary care do not incorporate the provider or population characteristics. The currently used STAR-PU measure only adjusts for the age and gender composition of the NI population. Our results indicate the need for further adjustment for other factors to ensure unbiased comparison between GP practices, and to target interventions appropriately. Future research will focus on conducting an individual-level analysis by linking individual clinical, microbiology, and prescribing records to assess the extent to which prescribing conformed to local guidelines. Specifically, we will assess how the rates of antibiotic prescription deviate from the prescribing guidelines, based on the type of recorded infection.

## Figures and Tables

**Table 1:** Total prescriptions and the number of patients registered with a GP, with year-on-year percentage change.

Year	Prescriptions	% Change in Prescriptions	Registered Patients	% Change in Registered Patients
2014	1,564,707	-	1,855,949	-
2015	1,536,909	−1.8	1,873,675	1.0
2016	1,562,211	1.6	1,902,438	1.5
2017	1,485,830	4.9	1,926,107	1.2
2018	1,438,796	−3.2	1,953,334	1.4
2019	1,421,520	−1.2	1,982,733	1.5
2020	1,155,323	−18.7	1,999,095	0.8
**Total**	**10,165,296**			

**Table 2:** Summary statistics for the standardised number of antibacterial drugs (per 1000 registered patients).

	2014	2015	2016	2017	2018	2019	2020
median (IQR)	843.1 (746.3–952.2)	816.0 (727.1–936.2)	819.8 (724.4–933.7)	768.7 (674.5–877.3)	723.5 (643.8–844.3)	709.1 (624.6–811.1)	573.0 (498.4–670.3)
min	308.6	297.9	313.4	282.8	269.6	275.4	199.2
max	1444.8	1439.6	1391.9	1217.5	1194.3	1092.1	926.5

## Data Availability

Publicly available datasets were analysed in this study. This data can be found here: https://hscbusiness.hscni.net/services/3178.htm (accessed on 20 November 2021).

## Supplementary Materials

The following supporting information can be downloaded at: https://www.mdpi.com/article/10.3390/antibiotics11010017/s1, Table S1: The standardised number of prescriptions by antibiotic class in the period 2014–2020.
