# Supplementary material for: Trends, Variation, and Factors Influencing Antibiotic Prescribing: A Longitudinal Study in Primary Care Using a Multilevel Modelling Approach
Source: Antibiotics (Basel). 2021 Dec 24;11(1):17. doi: 10.3390/antibiotics11010017 (PMC8772723; doi:10.3390/antibiotics11010017)
Supplement: Supplementary file 1 [file antibiotics-11-00017-s001.zip › antibiotics-1486144-supplementary.pdf]

Supplementary Material

Table S1. The standardized number of prescriptions by antibiotic class in the period 2014-2020.

	**2014**	**2015**	**2016**	**2017**	**2018**	**2019**	**2020**
Penicillins	442.4	427.6	424.9	389.4	372.2	357.6	265.4
Cephalosporins and other beta-lactams	40.1	38.5	35.6	32.6	31.1	30.4	31.2
Tetracyclines	97.0	99.7	104.1	102.7	101.5	103.1	88.4
Aminoglycosides	0.3	0.3	0.4	0.5	0.2	0.4	0.4
Macrolides	104.2	100.7	98.6	88.7	80.6	75.6	56.9
Clindamycin and lincomycin	0.9	1.1	1.2	1.4	1.4	1.2	1.1
Some other antibacterials	3.0	3.2	3.5	3.8	4.1	4.4	4.5
Sulfonamides and trimethoprim	85.3	82.6	82.6	80.1	75.8	72.0	64.6
Antituberculosis drugs	2.0	2.0	1.9	2.0	1.8	1.8	1.7
Antileprotic drugs	1.7	1.7	1.6	1.7	1.8	1.9	1.9
Metronidazole, tinidazole and ornidazole	12.1	11.7	11.4	11.1	10.6	10.3	9.5
Quinolones	19.3	17.9	17.3	16.1	14.5	12.1	10.1
Urinary-tract infection drugs	44.2	44.2	46.1	45.1	44.3	46.7	47.1
